# Rosemary‐Mediated Green Synthesis of ZnO Nanoparticles and their Integration into Hydrogel Matrices: Evaluating Effects on Wheat Growth and Antibacterial Properties

**DOI:** 10.1002/gch2.202400120

**Published:** 2024-07-19

**Authors:** Yağmur Uysal, Zeynep Görkem Doğaroğlu, Zehranur Çaylali, Delil Sefkan Karakulak

**Affiliations:** ^1^ Engineering Faculty Environmental Engineering Department Mersin University Mersin Turkey

**Keywords:** antibacterial potential, chlorophyll, germination, rosemary, wheat

## Abstract

In this study, the impact of zinc oxide nanoparticles (ZnO‐NPs) generated using rosemary extract, synthesized using environmentally friendly processes and integrated into a cross‐linked polymer matrix, on growth performance of wheat is evaluated. Rosemary extract used as coating, stabilizing, and reducing agents in this green synthesis method. Fourier transform infrared spectroscopy analyses demonstrated the presence of phytochemical constituents of the plant extract that served as capping agents during the synthesis process. The nanoparticles are sprayed to the plant leaves. The effects of nanoparticles within the hydrogel on plant development are compared with the effects of nanoparticles in suspension. The percentage of seed germination is unaffected by either rosemary‐ or raw‐ZnO‐NPs; however, the root and shoot elongation are considerably impacted by the nanoparticle treatments. The threshold concentrations are determined as 3000 mg L^−1^ for rosemary‐ZnO‐NPs and 2000 mg L^−1^ for raw‐ZnO‐NPs. Additionally, antibacterial test results showed that the activity level on Escherichia coli is higher for rosemary‐ZnO‐NPs compared to raw‐ZnO‐NPs. The results of this research may provide guidance on how green synthesis methods and the use of nanoparticle‐hydrogel composites in plant breeding can be used in future agricultural applications. This can be considered an important step in terms of agricultural innovations and sustainability.

## Graphical abstract

The effects of R‐ZnO‐NPs (rosemary plant extract mediated ZnO‐NPs), and Raw‐ZnO‐NPs by foliar and soil applications (their hydrogel forms) on the growth parameters of wheat seedlings are investigated. The growth parameters do not different from the control for foliar and hydrogel treatment. The plant biomass and Zn uptake by wheat is higher than control. Antibacterial tests on *E. coli* and *S. aureus* showed that gram‐negative strains are more sensitive to R‐ZnO‐NPs than gram‐positive.

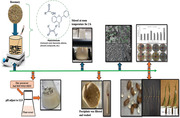

## 1 Introduction

Due to its novel uses in the fields of medicine, biology, sports equipment, electronics, solar energy, magnetics, and other industries, metal oxide nanoparticles, are receiving a lot of attention.^[^ ^1^ ^]^ One of the most promising semiconductor materials for use in a variety of applications is zinc oxide (ZnO). Due to the expansion of nanotechnological developments and study, zinc oxide nanoparticles (ZnO‐NPs) with a large band gap (3.37 eV) and high binding energy (60 meV) are currently attracting a lot of attention. It has a wide range of applications in areas like environmental applications, chemistry, physics, biology, medicine, and electronics because of its inexpensive cost, non‐toxicity at low concentrations, chemical stability, and ability to change its optical and electrical properties.^[^ ^2^ ^]^ The function of zinc oxide nanoparticles (ZnO‐NPs) in plants and agriculture has garnered a lot of attention in recent years. Numerous benefits of NPs have been shown, and this beautiful material can replace numerous fertilizers, micronutrients, fungicides, and antimicrobial agents.^[^ ^3^ ^]^ Due to their biocompatible structure, potential antibacterial effects, and desirable optical, electrical, and photocatalytic properties, ZnO‐NPs stand out among metallic nanoparticles.^[^ ^4^, ^5^ ^]^ This demonstrates how ZnO‐NPs are used in a variety of fields, such as chemical sensors, solar cells, and photocatalysis.^[^ ^3^ ^]^

ZnO‐NPs can be made using a variety of simple processes. Wet chemical processing, hydrolysis/condensation, and sol‐gel processing are the most frequently used techniques.^[^ ^6^ ^]^ While generating nanoparticles, the procedures and conditions utilized can easily change the physical and chemical behavior of ZnO‐NPs. The majority of these synthesis methods are pricey and demand exacting experimental conditions (such as pressure, temperature, energy, and time). Most importantly, these methods involve dangerous chemicals that endanger ecosystem viability and aggravate environmental degradation, which is currently the most urgent problem.^[^ ^7^ ^]^ Green synthesis of ZnO‐NPs is gaining a lot of interest at the moment due to a number of its advantages over traditional methods, including being quicker, less expensive, and more environmentally friendly. Microorganisms, plant biomass, seeds, leaves, and bark have recently emerged as the most feasible and environmentally secure way to make nanoparticles.^[^ ^8^ ^]^ In light of their low prices, non‐toxicity, environmental compatibility, biocompatibility, and use of nontoxic stabilizers, green or eco‐friendly synthesis techniques have been created.^[^ ^9^ ^]^ The most promising synthesis technique is the use of plant extracts in “green synthesis,” which can be accomplished by using plant extracts or specific microorganisms (algae, fungi, bacteria, etc).^[^ ^10^, ^11^ ^]^ Because of their accessibility, capacity for large‐scale biosynthesis, ability to preserve metabolites, and handling safety, plant extracts are one of the many biosynthesis methods that can be used to create nanoparticles.^[^ ^12^ ^]^ Moreover, plant extracts serve as stabilizers and capping agents to restrain the growth of the crystals and stop them from aggregating.^[^ ^13^ ^]^ Alamdari et al. claim that functional groups of compounds give away electrons, changing Zn^2+^ into Zn^+^.^[^ ^14^ ^]^ The hydroxyl and phenolic compounds in the leaf extracts are responsible for the formation of NPs by converting Zn^2+^ to Zn^+^ ions.^[^ ^15^ ^]^ Ezealisiji et al. made similar claims, indicating that zinc nitrate was ionized in an aqueous media to generate Zn^2+^, which was then reduced to Zn^+^ by a phytochemical present in the extract.^[^ ^16^ ^]^ The hydroxyl group in the polyphenols may convert to zinc hydroxide during the hydrolysis process.^[^ ^17^ ^]^

To date, many plant and fruit extracts have been employed to create a variety of metallic nanoparticles such as *Parthenium hysterophorus*,^[^ ^18^ ^]^ *Citrus aurantifolia*,^[^ ^19^ ^]^ *Hibiscus subderiffa*,^[^ ^12^ ^]^ *Mussaenda frondosa*,^[^ ^2^ ^]^ *Rubus fairholmianus*,^[^ ^20^ ^]^ *Thymus syriacus*,^[^ ^21^ ^]^ *Myrica esculenta*,^[^ ^5^ ^]^ and *Syzygium cumini*.^[^ ^22^ ^]^ It is clear that there has been a rise in interest in metal oxide nanoparticles made using the green synthesis technique in recent years in order to provide a potent antibacterial effect. Studies have also demonstrated the powerful bactericidal effects of ZnO‐NPs produced through green synthesis on a range of bacteria.^[^ ^5^, ^10^ ^]^ The main mechanism at work in this instance is that the nanoparticles produced through green synthesis come into touch with the receptors on the surface of the microorganisms' cell walls, resulting in an antibacterial action. To the best of our knowledge, no studies have compared raw and green produced ZnO‐NPs with plant extracts such *Rosmarinus officinalis* L. (rosemary). A blooming plant in the *Lamiaceae* family, *Rosmarinus officinalis* is most frequently referred to as rosemary. It is a perennial herb that can grow fairly large and keep its beauty for many years. The leaves are needle‐like and everlasting. Rosemary is frequently grown as an ornamental plant because it may be able to ward off pests. In addition to being used to flavor food, rosemary is also utilized to cure a number of illnesses.^[^ ^23^ ^]^ As a result, in the current investigation, we sought to develop a straightforward and long‐lasting method for the synthesis of ZnO‐NPs employing rosemary plant extracts as capping, stabilizing, and reducing agents.

Due to its intriguing biocompatibility and adjustable physicochemical features, hydrogel, characterized by a hydrophilic nature and a 3D network structure, has garnered considerable attention across various domains.^[^ ^24^ ^]^ Regrettably, conventional hydrogels suffer from poor mechanical performance owing to the lack of efficient energy dissipation mechanisms and multifunctional hydrogel components, imposing significant limitations on their utility.^[^ ^25^ ^]^ In recent times, diverse microstructures and crosslinking methods have been employed in the creation of hydrogels, including polyelectrolyte (PEC) hydrogels,^[^ ^26^, ^27^ ^]^ nanocomposite (NC) hydrogels,^[^ ^28^ ^]^ and hybrid double network (HDN) hydrogels.^[^ ^29^ ^]^ Nanocomposite hydrogels, which amalgamate hydrogels with nanoscale materials, represent hybrid materials harnessing the advantages of both components.^[^ ^30^ ^]^ The incorporation of nanoparticles elevates the mechanical performance of raw hydrogels, imparting distinctive properties such as electrical conductivity, magnetism, antimicrobial activity, and optical capabilities.^[^ ^31^, ^32^, ^33^, ^34^ ^]^ Nanoparticle network (NN) hydrogels, a subtype of NC hydrogels, emerge when nanoparticles play a pivotal role in crosslinking the bulk hydrogel network.^[^ ^35^ ^]^ In contrast to conventional hydrogels, NN hydrogels leverage nanoparticles as multifunctional cross‐linkers, streamlining hydrogel formation, thereby eliminating the need for additional cross‐linkers with multiple polymerizable functional groups.^[^ ^36^, ^37^ ^]^ The incorporation of a diverse array of nanoparticles with varying sizes, shapes, and functionalities directly into the network enables NN hydrogels to assume versatile morphologies and exhibit multiple activities.^[^ ^38^, ^39^ ^]^ Consequently, NN hydrogels find widespread applications across numerous fields.

In this investigation, hydrogel networks were developed by integrating polyvinyl alcohol/sodium alginate (PVA/SA), acting as a nanoparticle cross‐binder, into a polymeric matrix consisting of polymeric alcohol. The resulting hydrogels, formed through the binding of ZnO‐NPs generated via green synthesis, were assessed within the wheat growth environment to examine their impact on seed germination, as well as plant growth and development. The primary objective of our research is to devise an uncomplicated and sustainable protocol for ZnO‐NPs production, utilizing herbal rosemary extract as multifunctional agents—serving as capping, stabilizing, and reducing agents.

We meticulously searched into the structural and morphological properties of the obtained ZnO‐NPs using standard characterization techniques. Furthermore, our investigation investigated the influence of ZnO‐NPs derived from rosemary extract on the growth factors and chlorophyll content of wheat (*Triticum aestivum* – İkizce 96). The nanoparticles were applied by spraying method, while simultaneously placing them in the plant's root zone. The comprehensive evaluation of their effects on plant growth was conducted, and concurrently, nanoparticles were incorporated into the hydrogel. We explored how the hydrogel‐NP composite impacted plant development and growth factors, comparing it to the presence of the nanoparticle in powder form within the plant's root zone. To ascertain the plant's uptake of nanoparticles, acid dissolution was performed on the plants, and the Zn contents were determined.

This all‐encompassing study aims to furnish valuable insights into the green synthesis of ZnO‐NPs using plant extracts and the collaborative utilization of these nanoparticles with hydrogels, shedding light on their effects on plant growth. The results of this research may provide guidance on how green synthesis methods and the use of nanoparticle‐hydrogel composites in plant breeding can be used in future agricultural applications. This can be considered an important step in terms of agricultural innovations and sustainability. This research presents a novel approach to synthesizing ZnO nanoparticles using rosemary extract, providing a sustainable alternative to conventional methods. The study uniquely demonstrates the dual benefits of these nanoparticles in promoting plant growth and offering antibacterial properties, highlighting their potential for agricultural applications. This environmentally friendly synthesis method reduces the ecological footprint and enhances the bioactivity of ZnO‐NPs, paving the way for innovative uses in crop protection and growth enhancement.

## 2 Results and Discussion

### 2.1 Characterizations of Synthesized ZnO‐NPs Powders

**Figure 1:**
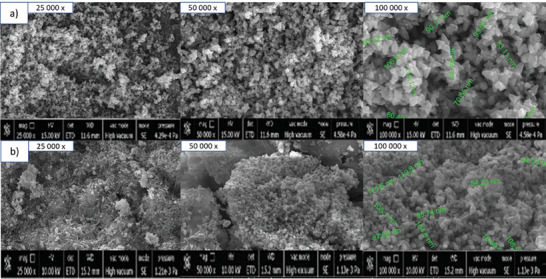
SEM images of a) R‐ZnO‐NPs, b) Raw ZnO‐NPs.

ZnO‐NPs' surface morphology was identified and displayed in **Figure** **1**. In the SEM pictures, it was possible to see the clear particles of ZnO‐NPs with a triangle‐like structure that were created by the rosemary extract. Particles are uniformly distributed in SEM pictures. The created ZnO‐NPs occasionally exhibited significant aggregation development, which is typical of nanoparticles manufactured by green synthesis. This is because biosynthetic NPs have a larger surface area and cluster or gather together due to their persistent affinity.^[^ ^40^ ^]^ Agglomeration of ZnO nanoparticles can occur due to several factors primarily related to physical and chemical interactions between the nanoparticles and the surrounding medium. The polarity of the nanoparticles and their electrostatic attraction led to particle agglomeration.^[^ ^41^ ^]^ ZnO nanoparticles have a high surface‐to‐volume ratio, leading to high surface energy, which causes them to aggregate to minimize this energy. Attractive van der Waals forces between nanoparticles also contribute to agglomeration, as these forces are significant at the nanoscale where particles are very close to each other. Inadequate stabilization mechanisms, such as insufficient capping agents or surfactants, can fail to prevent agglomeration; in this study, if the concentration or effectiveness of rosemary extract, acting as a capping and stabilizing agent, is insufficient, it may not fully prevent agglomeration. The polarity of the nanoparticles and their electrostatic attraction led to particle agglomeration. The pH and ionic strength of the medium influence nanoparticle stability, where changes in pH can alter surface charges leading to reduced electrostatic repulsion, and high ionic strength can screen these charges, allowing nanoparticles to come closer and agglomerate. The number of H^+^ and OH^−^ ions changes as the pH value rises, which affects the structure, shape, and production of ZnO. At high pHs, smaller nanoparticles also develop. Because there are more OH^−^ ions present, they are more strongly attracted to the positively charged Zn^2+^, which stimulates the creation of strong Zn‐O bonds in the structure.^[^ ^42^ ^]^ Additionally, during the drying process, solvent evaporation can bring nanoparticles into close proximity, causing agglomeration due to capillary forces. Mechanical stirring or mixing during synthesis or application processes can also lead to physical agglomeration as nanoparticles collide with each other. The basicity of the solution can be changed to customize different ZnO morphologies, such as rods and flowers, claim.^[^ ^43^ ^]^ R‐ZnO‐NPs had an average particle size of 88.87 nm in SEM images (Figure 1a) and Raw‐ZnO‐NPs had an average particle size of 98.53 nm (Figure 1b). The size measurement results showed that the average grain size of Raw‐ZnO nanoparticles was higher than the nanoparticles produced by green synthesis.

**Figure 2:**
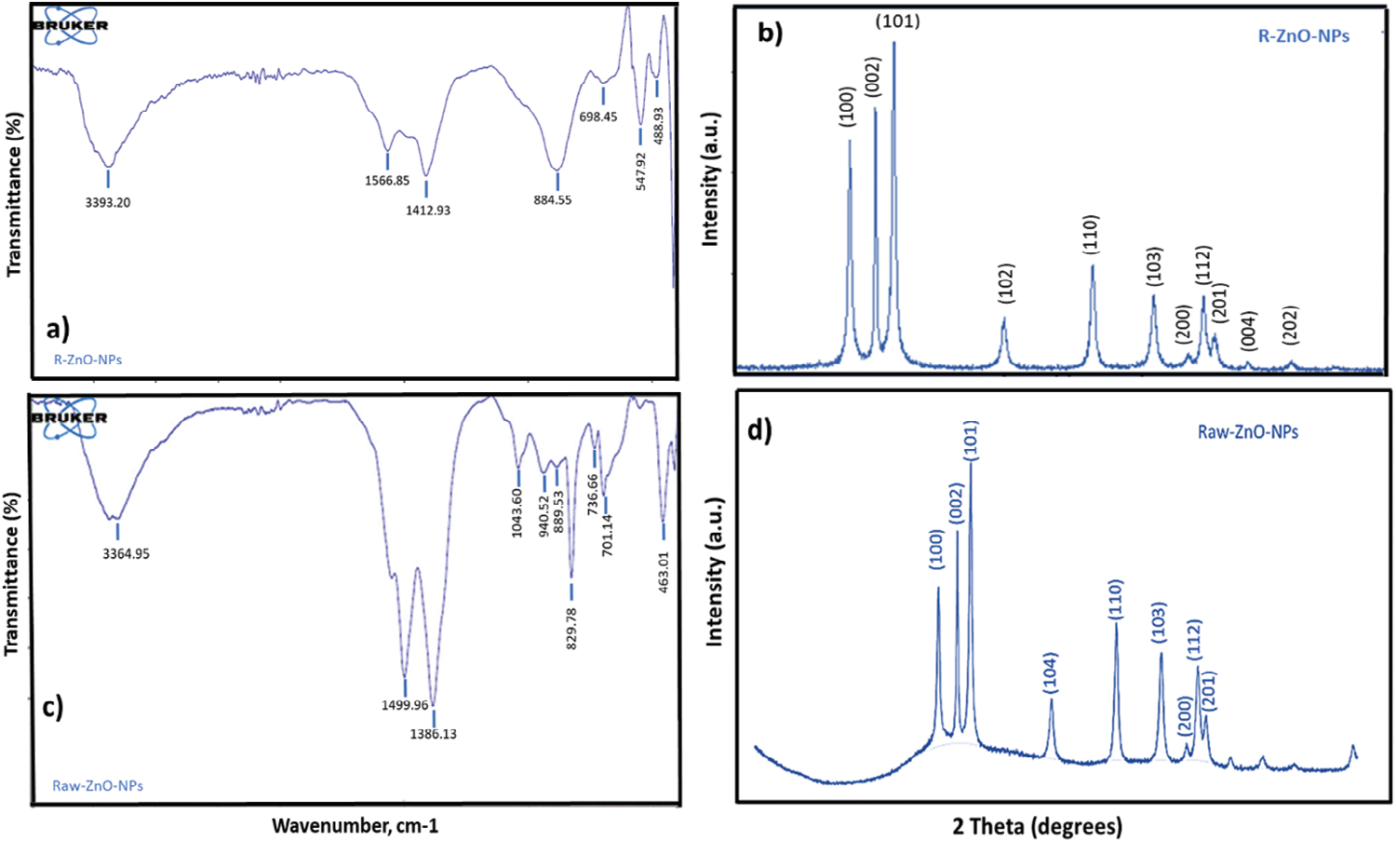
FTIR spectra and XRD diffractograms of a,b) green synthesized ZnO‐NPs using rosemary extract (R‐ZnO‐NPs), and c‐d) Raw‐ZnO‐NPs.

In order to identify the numerous distinctive functional groups connected to the produced nanoparticles, FTIR research was performed on ZnO‐NPs. FTIR analyses between 4000 and 400 cm^−1^ were used to describe and identify the biomolecules responsible for the synthesis of R‐ZnO‐NPs and Raw‐ZnO‐NPs (**Figure** **2**). It is known as a complex generation of Zn^+^‐polyphenols in the reaction solution because Zn^2+^ ions from zinc acetate associated with polyphenols present in the plant extract and reduced to Zn^+^ during the synthesis of ZnO‐NPs. Similar peaks in the FTIR spectra of green produced ZnO‐NPs from plant extracts showed these complex phenomena (Figure 2a,c).

**Figure 3:**
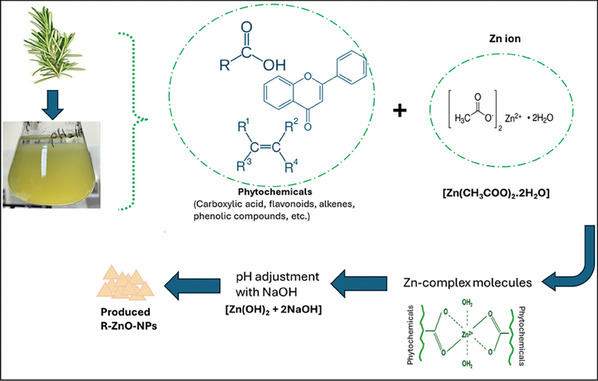
A schematic illustration of proposed mechanism of ZnO NPs formation with green approach.

The FTIR spectrum of the R‐ZnO‐NPs (Figure 2a) showed absorption bands at 3393, 1566, 1412, 884, 698, 547, and 488 cm^−1^. The presence of the hydroxyl functional groups of alcohols and phenolic compounds that may be present in plant extracts could be the cause of the broad and extremely strong bands at 3393 cm^−1^. The aromatic rings in polyphenolic compounds have C═C stretching vibrations, and this is related to the peak at 1566 cm^−1^. The peak at 1412 cm^−1^ results from symmetrical acids' COO‐stretching mode.^[^ ^44^ ^]^ The main absorption band of ZnO lies between 400 and 600 cm^−1^.^[^ ^45^, ^46^ ^]^ The ZnO vibration is associated to strong band at 547 cm.^−1[^ ^47^ ^]^ The absorption at 884 cm^−1^ is due to the formation of Zn's tetrahedral coordination. Similar to Raw‐ZnO‐NPs, bending vibrations of alkanes and alkenes (C═C bending) are observed at 829 cm^−1^. The detected peak in the 698 cm^−1^ represents the stretching vibrations of ZnO nanoparticles. The vibration mode associated with metal‐oxygen (ZnO stretching vibrations) corresponds to the absorption peak at 547 cm^−1^. The FTIR spectrum of the Raw‐ZnO‐NPs (Figure 2c) showed absorption bands at 3364, 1499, 1386, 1043, 940, 889, 829, 736, 701, 554, and 463 cm^−1^. The fundamental mode of vibration at 3364 cm^−1^ in the FTIR spectrum of the Raw‐ZnO‐NPs may be caused by O─H stretching and deformation, which are both attributed to the water adsorption on the metal surface. The peak at 1386 cm^−1^ is related to the asymmetric stretching vibration of C═O. The C─O stretching vibration is responsible for the binding at 1043 cm^−1^. The absorption at 889 cm^−1^ is due to the formation of Zn's tetrahedral coordination. The bending vibrations of alkanes and alkenes (C═C bending) are observed at 829 cm^−1^.^[^ ^48^, ^49^, ^50^ ^]^ The detected peaks in the range of 736 to 701 cm^−1^ represent the stretching vibrations of ZnO nanoparticles. The vibration mode associated with metal‐oxygen (ZnO stretching vibrations) corresponds to the absorption peak at 554 cm^−1^. Depending on the FTIR peaks, the proposed mechanism of ZnO NPs formation with green approach was given in **Figure** **3**.

The hexagonal wurtzite structure of ZnO nanoparticles is characterized by these peaks (JCPDS card number: 36–1451).^[^ ^51^ ^]^ Figure 2b,d depict the XRD diffractograms of the prepared green ZnO photocatalysts made of R‐ZnO‐NPs and Raw‐ZnO‐NPs. Figure 2b,d showed distinct peaks in the XRD patterns at 2θ = 31.76°, 34.40°, 36.20°, 47.52°, 56.58°, 62.83°, 66.36°, 67.93°, 69.07°, 72.52°, and 76.94° that match well with planes Miller indices (100), (002), (101), (102), (110), (103), (200), (112), (201), (004), and (202) for R‐ZnO‐NPs.^[^ ^51^ ^]^ The material's nanoscale particle size is confirmed by the noticeable line broadening of the XRD peaks. Besides that, the crystallite size of the (101) plane, determined using the Debye‐Scherrer formula, was found to be 21.87 nm R‐ZnO‐NPs and 26.78 nm for Raw‐ZnO‐NPs, further corroborating the nanoscale dimensions of the synthesized ZnO‐NPs.

The high crystallinity and purity of the generated ZnO‐NPs were respectively substantiated by the prominent diffraction peaks and the absence of impure reflections. ZnO produced through green synthesis was entirely pure and extraordinarily crystalline in its unaltered original state, and no peaks associated with contaminants were found. These results support earlier findings.^[^ ^48^, ^52^ ^]^ In contrast to past studies,^[^ ^53^, ^54^ ^]^ we carried out the experiment at ambient temperature rather of using high temperatures. This suggests that highly crystalline nanomaterial could be produced using a quick, simple, and environmentally benign technique of synthesis. As a result, our study has proven to be a better and more practical option for synthesizing ZnO‐NPs in terms of energy efficiency.

### 2.2 Characterization of ZnO‐NPs‐Doped Hydrogels

**Figure 4:**
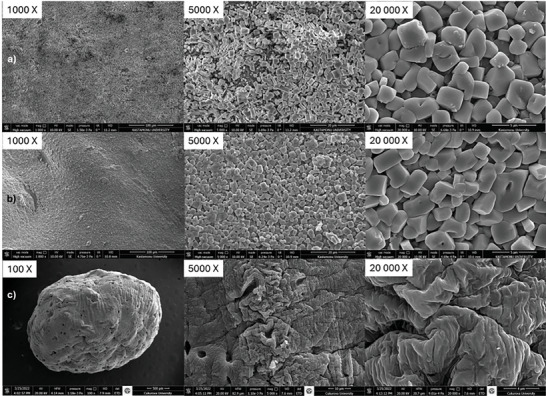
SEM images of synthesized a) R‐ZnO‐HG, b) Raw‐ZnO‐HG, c) Raw‐HG.

SEM studies (**Figure** **4**a,b) reveal that the R‐ZnO‐HG and Raw‐ZnO‐HG materials form morphological structures of approximately equal sizes, characterized by tile‐like cubic structures surrounded by a polymeric material coating. In these images, ZnO nanoparticles formed a core structure. However, Raw‐HG exhibits a curved and perforated (porous) structure (Figure 4c). The cubic forms of ZnO filled the surface of the hydrogel by covering these folded structures.

**Figure 5:**
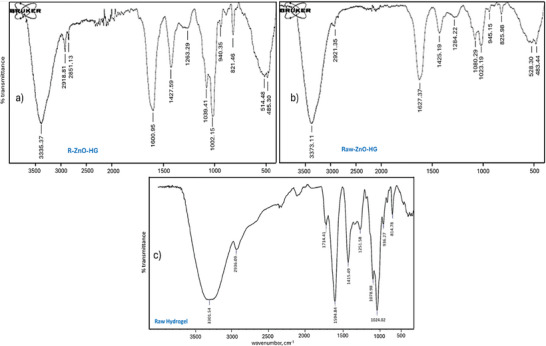
FTIR spectra of a) R‐ZnO‐HG, b) Raw‐ZnO‐HG, and c) Raw‐HG.

The FTIR analysis revealed that intermolecular interactions played a crucial role in influencing the vibration of functional groups within the fragments of the superabsorbent hydrogel (**Figure** **5**). The OH^−^ stretching vibrations in two types of hydrogels were associated with the FTIR peaks observed at 3375 and 3373 cm^−1^, as reported in previous studies.^[^ ^55^, ^56^, ^57^ ^]^ The presence of SA led to a significant broadening of the hydroxyl stretch bands, providing strong evidence for the formation of hydrogen bonds between the hydroxyl groups of PVA and SA. C─H bending of alkanes were evident at 1500 cm^−1^. The medium‐intensity peaks at 1427 cm^−1^, representing the C═O stretching band, exhibited characteristics of both PVA and SA, indicating a potential hydrogen bond formation between the hydroxyl groups of PVA and the SA group in the hydrogels (Figure 5a). The weak and broad peaks at 1263 and 1284 cm^−1^ indicates C═O stretching.^[^ ^58^ ^]^ Aromatic C‐H stretching vibrations groups were evident in the FTIR spectra at 1079 and 1080 cm^−1^.^[^ ^59^, ^60^ ^]^ Another noteworthy peaks at 1022 and 1023 cm^−1^ were attributed to the C─O─C stretching bond from sodium alginate in the hydrogel.^[^ ^61^ ^]^ The small and sharp peaks at 940 cm^−1^ reveals the C═C bending. In the R‐ZnO‐HG FTIR spectra, there is a weak peak at 881 cm^−1^ indicating the C─H bending. Hydrogen‐bonding C─OH groups were evident in the FTIR spectra at and 821 cm^−1^.^[^ ^55^, ^59^, ^62^ ^]^ Zn‐O in Zn(OH)_2_ appeared as a broad band at 514 and 528 cm^−1^. Furthermore, C═O stretching bonds derived from sodium alginate were observed at 485 and 483 cm^−1^.^[^ ^61^ ^]^

### 2.3 Swelling Properties of Hydrogels

**Figure 6:**
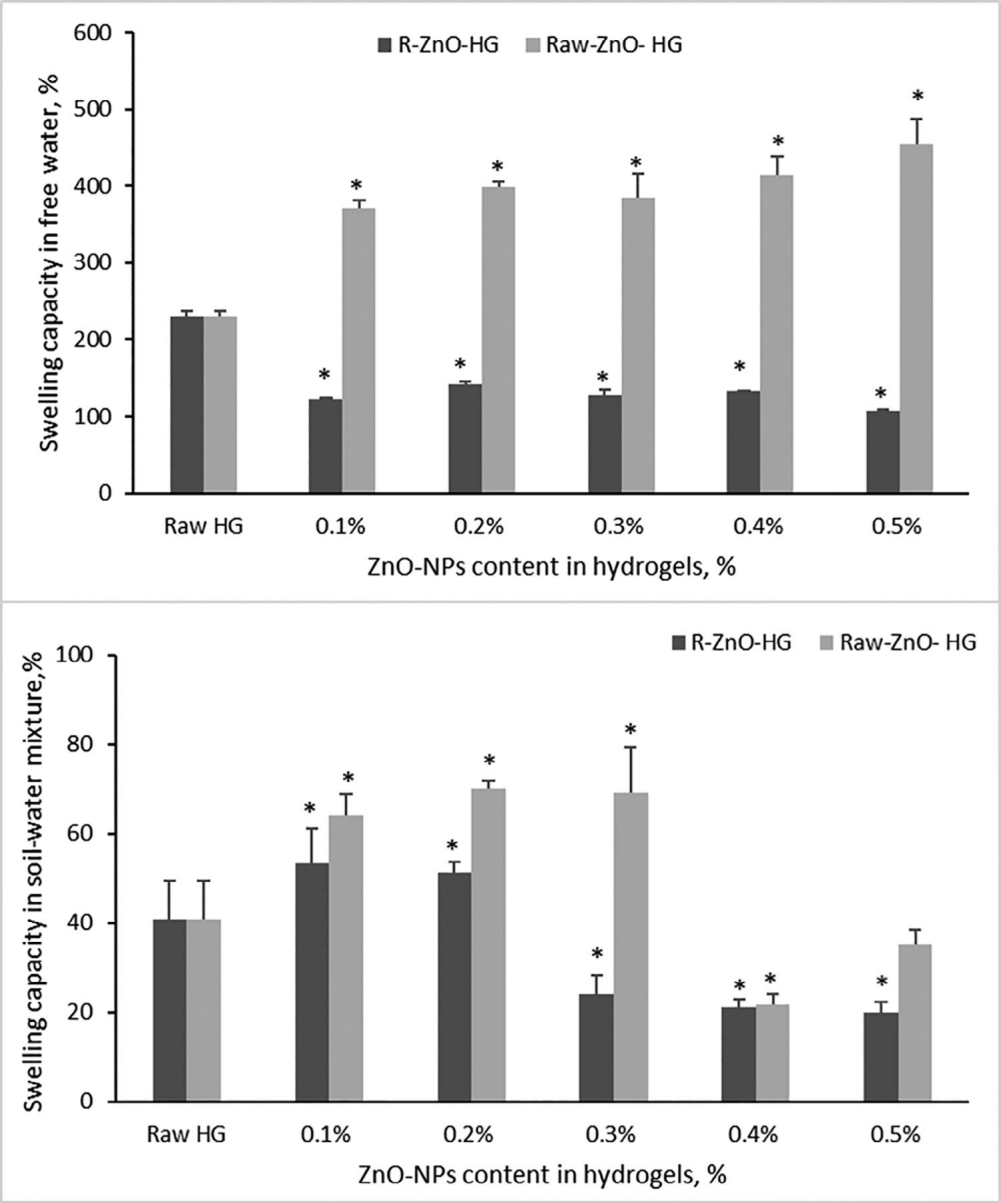
Swelling capacity of R‐ and Raw‐ZnO‐HGs a) in free water and b) in soil‐water mixture (* means there was a significant effect compared to Raw HG as control, p < 0.05).

The water‐holding capacity thus swelling capacity is the most important parameter for hydrogels in agricultural applications. The swelling capacities of the synthesized hydrogels (Raw‐HG, R‐ZnO‐HG, and Raw‐ZnO‐HG) in free water and in soil‐water mixture were determined. The swelling capacity of synthesized hydrogel depended on the content of ZnO‐NPs in hydrogels, as well as the swelling media. Results showed that the addition of ZnO‐NPs to hydrogels significantly improved the swelling capacity, compared to Raw‐HG (p < 0.05). Results showed that, the Raw‐ZnO‐NPs doping in hydrogels more enhanced the swelling capacity than R‐ZnO‐NPs. Besides that, the maximum swelling capacity was determined at the content of 0.5% Raw‐ZnO‐NPs in hydrogel as 453.9% (**Figure** **6**a). However, the optimum swelling capacity was determined at the content of 0.2% Raw‐ZnO‐NPs‐HG, as like R‐ZnO‐HGs. The maximum swelling capacity of R‐ZnO‐HGs was determined at this concentration as 141.5%. Meanwhile, the Raw‐HGs swelling capacity were determined as 230.2%. The effective mechanisms in swelling capacity of hydrogels were hydration forces, capillary, and osmotic pressure, due to water and polymers interaction,^[^ ^63^, ^64^ ^]^ thus the water molecules can penetrate the pores on the surface of hydrogels.^[^ ^65^ ^]^ Briefly, polar groups, which gain hydrophilic properties by preserving their physical and chemical strength properties, swell with this interaction.^[^ ^64^ ^]^ In addition, the Raw‐ZnO‐HGs and R‐ZnO‐HGs showed a good swelling capacity in soil‐water mixture, especially at low concentrations of ZnO‐NPs, compared to Raw‐HGs (Figure 6b) (p < 0.05). Results proved that the addition of ZnO‐NPs to hydrogels improved the swelling capacity. However, the swelling capacities in soil‐water mixture were lower than in free water, as like report of.^[^ ^66^ ^]^ The maximum swelling capacity in soil‐water mixture was determined at the content of 0.2% Raw‐ZnO‐HG as 70.1%. Meanwhile, the maximum swelling capacity of R‐ZnO‐HG was determined at the 0.1% R‐ZnO‐NPs content as 53.3%. These results were similar with the study of.^[^ ^67^ ^]^

### 2.4 Germination Tests

**Figure 7:**
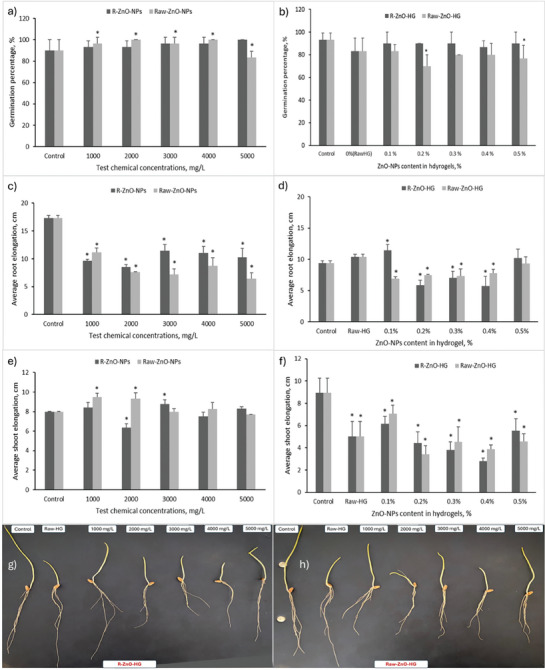
Effects of R‐ZnO‐NPs, Raw‐ZnO‐NPs, R‐ZnO‐HG, and Raw‐ZnO‐HG on a,b) germination percentage, c,d) root elongation, and e,f) shoot elongation of wheat (* means there was a significant effect compared to control, p < 0.05), and g,h) images of wheat seedlings under treatment of hydrogels.

The seed germination tests were conducted to determine the principle phytotoxicological effects of green synthesized R‐ZnO‐NPs. The germination tests are used to evaluate seed viability^[^ ^68^ ^]^ and response to biotic and abiotic stress factor. Results showed that the seed germination percentage of wheat increased with increasing R‐ZnO‐NPs (p>0.05) and Raw‐ZnO‐NPs (p < 0.05) concentrations compared to control (**Figure** **7**a). The minimum and the maximum seed germination percentages were determined in 4000 mg L^−1^ Raw‐ZnO‐NPs as 100% (p < 0.05) and in 5000 mg L^−1^ R‐ZnO‐NPs (p>0.05) concentration as 100%, respectively. As observed in the Figure 7a, the Raw‐ZnO‐NPs showed the enhancing effects on seed germination, however there was a threshold to apply this nanoparticle to wheat seedlings. Raw‐ZnO‐NPs positively affected the seed germination of wheat at low concentrations. Besides that, the green synthesized ZnO‐NPs (R‐ZnO‐NPs) need high concentration to reach maximum germination percentage. The zinc (Zn) is a key element for vital activities in the living organisms, such as it constitutes all the enzymes such as transferases, oxidoreductases, and ligases and also has key role in photosynthesis and declining the photooxidation.^[^ ^69^ ^]^ Given all these benefits, it's obvious that the seeds will volunteer to uptake the Zn from synthesized nanoparticles. All these germination results showed the wheat seeds benefited from the R‐ and Raw‐ZnO‐NPs. Literature studies have stated that this benefit occurs through the penetration of nanoparticles into the seed coat pores (micropyles).^[^ ^68^, ^70^ ^]^ In the literature, there were differences between the germination results and applied green synthesized ZnO‐NPs types. It mostly dependent on the used plant extract, the morphological properties of the synthesized ZnO‐NPs and also the wheat species. There are little studies in the literature about the effects of green synthesized ZnO‐NPs on wheat seeds.^[^ ^68^, ^70^, ^71^ ^]^ Azmat et al. showed that the green synthesized ZnO‐NPs from papaya fruit extract help the seed germination of wheat against to external stress factors.^[^ ^72^ ^]^ The authors used the aloe vera extract to synthesizes of ZnO‐NPs and showed there were no phytotoxic effects on bread wheat seed germination.^[^ ^73^ ^]^ Besides that, the other studies showed green synthesized ZnO‐NPs used different plant extract positively affected the seed germination percentage of other plants.^[^ ^74^ ^]^

In the hydrogel treatment in germination process, result showed the germination percentage decreased with increasing Raw‐ZnO‐NPs content in hydrogels (Figure 7b). The germination percentage in control groups determined as 93%, while the R‐ZnO‐doped hydrogels germinated the wheat seeds as 90% at almost all concentrations. However, it was seen that the presence of R‐ZnO‐NPs in hydrogels positively affected the germination percentage, compared to Raw‐HG and Raw‐ZnO‐HG results. Presence of the Raw‐ZnO‐NPs in hydrogels had negative effects on germination percentage of wheat (Figure 7b). The minimum germination percentage was determined at the treatment of 0.2% Raw‐ZnO‐HG as 70% (p < 0.05). Doping hydrogels with different materials can change the effectiveness of these materials on plants. Of course, the type of plant applied is one of the most important factors here. Such as, reference^[^ ^75^ ^]^ showed the synthesized cellulose‐based hydrogels was the best media to maximum seed germination percentage for lettuce, however, it was not the best for the other seed types (*Brassica juncea*, *Ipomoea aquatica*, and *Solanum lycopersicum*). In addition to, reference^[^ ^76^ ^]^ synthesized corncob based semi‐IPN hydrogel for slow‐release nitrogen fertilizer with bentonite, and the authors showed the germination percentage of cotton plants increased with hydrogel treatments. Although, the other studies were not compared to doping hydrogels and raw hydrogels, doped hydrogels with different additives results were fit in with our results.

Although germination percentages are the first indicator of the response of plants to biotic or abiotic stress factors, a significant difference in this parameter may not always be observed. Therefore, the first evaluation after germination numbers/percentage is root and shoot elongation. Since the roots are the first plant limbs to come into contact with external factors, the roots usually show more pronounced reactions than the shoots. The vegetative growth (root‐shoot elongation, etc.) of plants helps to determine the beneficial threshold of nanoparticles.^[^ ^69^ ^]^ Some studies have proved that, although the higher concentrations of NPs had toxic effects on growth and development of wheat, the lower concentrations of NPs can positively affect.^[^ ^77^, ^78^, ^79^ ^]^ The effect of synthesized ZnO‐NPs on root and shoot length, of wheat seedlings is given in Figure 7c,d. There was visible inhibition in the growth rate of root at different ZnO‐NP types and concentrations compared to control (p < 0.05) (Figure 7c). The optimum concentration was determined for R‐ZnO‐NPs and Raw‐ZnO‐NPs as 3000 and 1000 mg L^−1^, respectively (Figure 7c). Results showed that the green synthesized R‐ZnO‐NPs were more beneficial than Raw‐ZnO‐NPs for the root elongation of wheat, but this is not enough to reach to control groups. The synthesized two types of ZnO‐NPs had negative effects on root elongation of wheat. However, these negative effects were not observed in the shoot elongation (Figure 7e). There were no adverse effects on shoot elongations almost all the types of ZnO‐NPs at high concentrations (4000–5000 mg L^−1^). The optimum shoot elongation was determined at the concentration of 1000 mg L^−1^ R‐ and Raw‐ZnO‐NPs. When root and shoot elongation are evaluated together, the optimum concentrations (threshold) that can be applied to wheat at the germination stage were found to be 3000 mg L^−1^ for R‐ZnO‐NPs and 1000 mg L^−1^ for Raw‐ZnO‐NPs. Some authors reported this threshold as 100 mg L^−1^ of green synthesized ZnO‐NPs using coriander plant extract^[^ ^74^ ^]^ and seaweed^[^ ^71^ ^]^ on tomato and maize seeds. This result proved that the beneficial effects of ZnO‐NPs were closely dependent on used plant extract.

Some differences were determined between the root and shoot elongation results obtained from hydrogel application and the results of suspended R‐ZnO‐NPs and Raw‐ZnO‐NPs application (Figure 7d,f). The root elongation reached the maximum value at the concentration of 0.1% R‐ZnO‐HG as 11.43 cm (p < 0.05), while the wheat seedlings treated with 0.5% Raw‐ZnO‐HG elongated 9.30 cm. However, contrary to expectations, the Raw‐HG (0% ZnO‐NPs) showed a good root elongation compared to ZnO‐NPs doped hydrogels, as 10.37 cm (Figure 7d). The shoot elongation also did not reach to control groups (Figure 7f). The maximum elongation obtained in the control groups. Besides that, it was determined at the 0.1% R‐ and Raw‐ZnO‐NPs treatment as 6.17 and 7.07 cm, respectively (p < 0.05). The results fit with the other studies in the literature.^[^ ^75^, ^80^, ^81^ ^]^

### 2.5 Pot Experiments

#### 2.5.1 Chlorophyll Content and Plant Height

**Figure 8:**
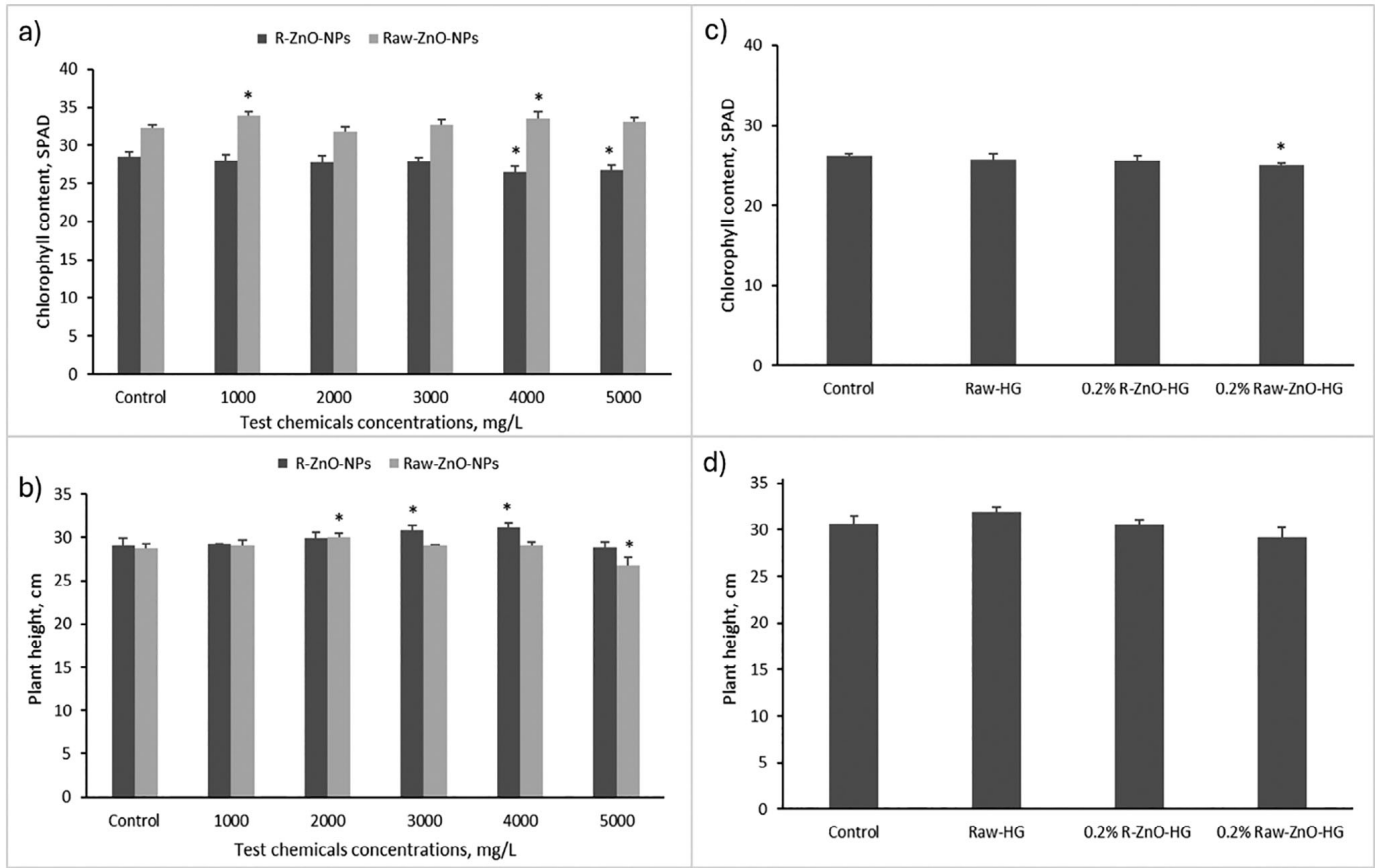
Effects of R‐ZnO‐NPs, Raw‐ZnO‐NPs, R‐ZnO‐HG, and Raw‐ZnO‐HG on a, c) chlorophyll content, b, d) height of wheat plants (* means there was a significant effect compared to control, p < 0.05).

Through foliar treatment, soil mixing, and/or seed priming, ZnO‐NPs are the most popular nanoparticles in current agriculture, yet they are less expensive than synthetic Zn fertilizers.^[^ ^82^ ^]^ They boost development and raise crop production and quality. For instance, 250–1000 mg L^−1^ of ZnO NPs applied to wheat, tomato, cabbage, and cauliflower boosted yield, pigment, and protein contents,^[^ ^83^, ^84^ ^]^ and improved growth and increased dry weight in *Cucumis sativus*.^[^ ^85^ ^]^ In this study, the effects of synthesized R‐ZnO‐NPs and Raw‐ZnO‐NPs by foliar treatment and also R‐ZnO‐HG, Raw‐ZnO‐HG and Raw‐HG by applying in soil on chlorophyll content, plant height, fresh, and dry weight of plants were determined. Results showed that the chlorophyll content of the wheat seedlings did not significantly change compared to control (**Figure** **8**a). The foliar treatment of R‐ZnO‐NPs caused decreases in chlorophyll content in wheat, especially at high concentrations (4000–5000 mg L^−1^) (p < 0.05). Figure 8a showed that the chlorophyll content of wheat slightly decreased with increasing R‐ and Raw‐ZnO‐NPs concentrations. The maximum chlorophyll content was measured at the 1000 mg L^−1^ R‐ZnO‐NPs (p < 0.05) and Raw‐ZnO‐NPs concentration, as 27.99 and 33.94 SPAD value, respectively. In the hydrogel application, the highest chlorophyll value was measured as 26.2 SPAD in the control group, while the lowest value was measured as 25.0 SPAD in the 0.2% Raw‐ZnO‐HG application (p < 0.05) (Figure 8b). The studies in the literature showed that the NP types and application method, used plants extract as stabilizing agent, and concentrations effected the photosynthetic pigments of wheat.^[^ ^86^, ^87^, ^88^, ^89^ ^]^

The plant height was significantly affected from the R‐ZnO‐NPs treatment, although, the Raw‐ZnO‐NPs treatment did not significantly affect compared to control, except 5000 mg L^−1^ Raw‐ZnO‐NPs concentration (p < 0.05) (Figure 8c). The maximum plant height of wheat was determined at the concentration of 3000 mg L^−1^ R‐ZnO‐NPs and 2000 mg L^−1^ Raw‐ZnO‐NPs as 31.1 and 30.1 cm, respectively. The hydrogel treatment also did not significantly affect the plant height (Figure 8d). The maximum plant height was determined in the Raw‐HG application. It was assumed that the water absorption and release properties of raw hydrogels was effective in this result. Although the maximum water absorption capacity in swelling properties was observed in ZnO‐doped hydrogels, water release may be high in raw hydrogels.

#### 2.5.2 Plant Biomass and Zn Uptake

**Figure 9:**
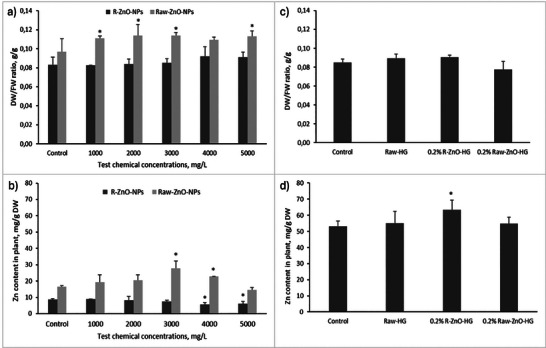
Effects of R‐ZnO‐NPs, Raw‐ZnO‐NPs, R‐ZnO‐HG, and Raw‐ZnO‐HG on a and c) the ratio of dry:fresh weight, b and d) Zn content in wheat plants (* means there was a significant effect compared to control, p < 0.05).

The ratio of dry weight (DW) to fresh weight (FW) was significantly affected from the treatment of synthesized Raw‐ZnO‐NPs (p < 0.05) (**Figure** **9**a). When the change of dry to fresh mass ratio of plants depending on the presence of ZnO‐NPs in the experimental media was examined, it was seen that R‐ and Raw‐ZnO‐NPs stimulated plant biomass slightly increase compared to control, especially at high concentrations (Figure 9a). The maximum increasement in plant biomass was determined at the 5000 mg L^−1^ R‐ and Raw‐ZnO‐NPs as 10% and 16.3%, compared to control. In the literature, ZnO‐NPs have been shown to increase shoot dry matter, leaf area, and seed weight per umbel when compared to controls.^[^ ^90^ ^]^ Higher values for seeded fruit per umbel, seed weight per umbel, and 1000‐seed weight were also noted.^[^ ^90^ ^]^ According to reference,^[^ ^91^ ^]^ treatments with ZnO‐NPs (25 nm mean particle size) at 1000 mg L^−1^ concentration enhanced seed germination, seedling vigor, and plant growth. Siddiqui et al., also reported that when plants were exposed to both concentrations of TiO_2_ or ZnO‐NPs, plant fresh weight, shoot dry weight, and root dry weight increased in comparison to the control plants^[^ ^92^ ^]^ In the hydrogel treatments, DW/FW ratio changed with hydrogel types, but it was not statistically significant (Figure 9b). The maximum increasement of biomass (DW/FW) was determined at the treatment of 0.2% R‐ZnO‐HG as 6.81%, compared to control. In addition to increase in the biomass was 5.56% for Raw‐HG and the decrease in biomass was determined in the treatment of Raw‐ZnO‐HG as 8.67%. In the hydrogel application, the increase in biomass is associated with the uptake of Zn by plants can be determined because the results are consistent with Figure 9d.

For plants and soil microorganisms that are helpful to plants, ZnO NPs are less harmful than other metal oxides.^[^ ^93^, ^94^ ^]^ Zinc is an important metal that has a key role in many metabolic processes, water uptake and transport, protein synthesis, and is found in essential enzymes such as isomerases, oxidoreductases, hydrolases, transferases, lyases, carbonic anhydrase, and ligases.^[^ ^87^, ^95^, ^96^, ^97^ ^]^ Therefore, the plants benefit from an increase in Zn level, which improved plant biomass. However, in some cases, especially at high concentrations, toxicity may be observed. This may result from disturbed intracellular balance as well as indirect effects on the uptake of other elements and inter‐element interactions. In the hydrogel applications, results showed that, the Zn uptake by wheat did not significantly change compared to control, except 0.2% R‐ZnO‐HG treatment (p < 0.05) (Figure 9d). Besides that, the foliar treatment showed the Zn uptake by wheat seedling significantly enhanced with the treatment of Raw‐ZnO‐NPs, while it was decreased with the treatment of R‐ZnO‐NPs (Figure 9c). The maximum Zn concentration was determined at the treatment of 3000 mg L^−1^ Raw‐ZnO‐NPs as 25.6 mg g^−1^ DW (p < 0.05) and this result also explains why the chlorophyll content was measured at high level in Raw‐ZnO‐NPs treatment.

### 2.6 Antibacterial Potential of Synthesized ZnO‐NPs

**Figure 10:**
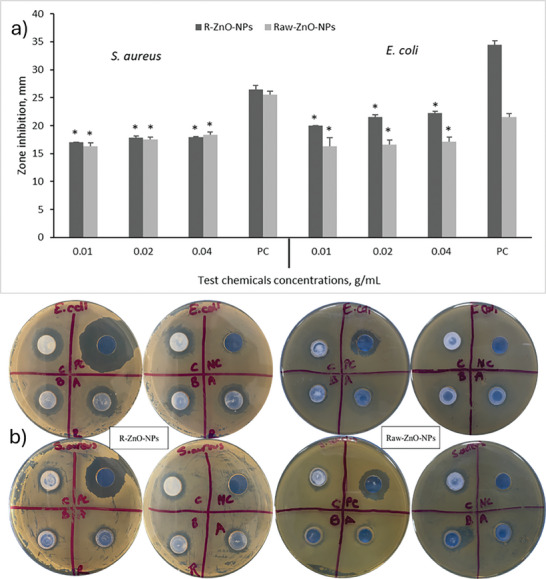
Antibacterial effects of R‐ZnO‐NPs and Raw‐ZnO‐NPs on *a) Staphylococcus aureus* and *Escherichia coli*, b) images of inhibition zones (* means there was a significant effect compared to positive control (PC), p < 0.05).

While the beneficial effect of Zn, a vital nutrient, on the plant is mentioned in the literature, its antibacterial properties should not be ignored.^[^ ^98^ ^]^ Metallic NPs have a potential antimicrobial role against bacterial species.^[^ ^99^ ^]^ Although NPs that can be designed as antimicrobial agents have many advantages, they also have disadvantages due to their effects that have not yet been identified and resolved. Studies have shown that nanomaterials, which have high surface area/volume ratio, unique physicochemical and promising antimicrobial properties, may reduce side effects, overcome resistance to antibiotics, increase overall pharmacokinetics through controlled drug release, and effective on infectious diseases by overcoming anatomical barriers (blood‐brain barrier) due to their material and particle size properties, unlike most drug molecules.^[^ ^100^, ^101^ ^]^ Although many studies have shown that antimicrobial NPs are more effective than component antibiotics alone, there are some challenges in translating this technology into clinical use. The most important unknown here is what its interactions with cells, tissues and organs are and the correct dose adjustment. In addition, the biggest problem is that the nanotoxicological effects are not fully known.^[^ ^101^ ^]^ Besides that, one of the most important factors in studies aiming to benefit from green nanobiotechnology in the development of sustainable, environmentally friendly and effective antimicrobial agents is the properties of the plant extract that will form the nanostructure.^[^ ^102^ ^]^ Phytochemicals (carboxylic acids, flavonoids, phenolic compounds, etc.) found in plant extracts already have many beneficial properties, and when they act as reducing agents in the production of nanomaterials, they form the basis for the biological property and toxicological properties of this material. For example, flavonoids and tannins have roles such as antioxidant and antimicrobial effects and thus combating reactive oxygen species.^[^ ^102^ ^]^ Therefore, the interaction of nanostructures with plant extracts is important in terms of increasing/improving their antibacterial potential. To determine the effectiveness of the synthesized R‐ and Raw‐ZnO‐NPs against to bacterial strains, i.e., *Staphylococcus aureus* as gram positive and *Escherichia coli* as gram negative via agar well diffusion method, the antibacterial tests were conducted. The concentrations of used ZnO‐NPs are the most important parameter in the formation of the inhibition zone. In this study, three different concentrations (0.01, 0.02, 0.04 g mL^−1^) were evaluated. Results showed that all the concentration of R‐ZnO‐NPs and Raw‐ZnO‐NPs effected on against to growth *E. coli* and *S. aureus* bacterial strains (**Figure** **10**a). The inhibition zone diameters of positive control (antibiotic) for the synthesized R‐ZnO‐NPs and Raw‐ZnO‐NPs were determined as 34.50 and 21.50 mm for *E. coli*, while it was 26.50 and 25.50 mm for *S. aureus*, respectively. In addition to, the percentages of maximum inhibition were observed at the 0.04 g/mL^−1^ concentration of nanoparticles. It was calculated as 64.54% (zone diameter: 22.27 mm for R‐ZnO‐NPs) and 79.84% (zone diameter: 17.17 mm for Raw‐ZnO‐NPs) for *E. coli* and 67.80% (zone diameter: 17.97 mm for R‐ZnO‐NPs) and 71.90% (zone diameter:18.33 mm for Raw‐ZnO‐NPs) for *S. aureus*. In one of the studies on this subject, the reference^[^ ^103^ ^]^ stated that green synthesized ZnO‐NPs using rosemary extract was more effective on *S. aureus* (19.2 mm) than on *E. coli* (12.7 mm). The authors’ result was opposite from this study. The differences may cause due to the synthesize method, because the authors applied calcination process. Besides that, the reference^[^ ^104^ ^]^ investigated the effects of rosemary supercritical fluid extract on gram‐positive and gram‐negative bacterial strains, and the authors showed the synthesized materials had strong antibacterial activity on both bacterial strains. On the other hand, the reference^[^ ^105^ ^]^ proved the antimicrobial effects can be enhanced with doping Ag ions to ZnO‐NPs synthesized with rosemary extract against to gram‐positive and – negative bacterial strains. In the literature there are many studies about the essential oil of rosemary, however, there is lack study that effects of synthesized ZnO‐NPs using rosemary extract. On the other hand, R‐ZnO‐NPs more effective on gram‐negative (*E. coli*) bacteria than gram‐positive (*S. aureus*). The gram‐positive bacteria are more sensitive to phytochemicals which are found in plants that protect plants against bacteria and responsible for scavenging toxic radicals (such as flavonoids, polyphenols, etc.).^[^ ^106^, ^107^ ^]^ Depending on this phenomenon, the effect mechanism of green synthesized R‐ZnO‐NPs can be explained. It was also determined that the ZnO‐NPs can be toxic on both gram‐positive and gram‐negative bacterial strains if there was sufficient nanomaterial in the growth media. This indicated that there was a threshold concentration in antibacterial tests, like the treatments in plant experiments. Some researchers investigated the effects mechanism of ZnO nanoparticles on the different gram‐positive and gram‐negative bacterial strains.^[^ ^10^, ^108^ ^]^ The authors explained that the bacterial growth inhibition depends on the cell membrane damage caused by nanoparticles, and/or electrostatic interactions of nanoparticles and bacteria.^[^ ^10^, ^107^, ^108^ ^]^ Bacterial cell damage, that is, the antibacterial effects of ZnO‐NPs, may also occur due to oxidative damage caused by hydroxyl radicals, hydrogen peroxide and superoxide radicals, and Zn^2+^ ions, which are reactive oxygen species released from the ZnO surface.^[^ ^101^, ^103^ ^]^ The large surface area of NPs and the antibacterial effects of Zn ions lead to increased production of these radicals.^[^ ^109^ ^]^ It has been stated in previous studies that hydrogen peroxide released from the ZnO surface can penetrate the cell membrane and therefore reduce/stop bacterial growth.^[^ ^103^ ^]^

## 3 Conclusion

This comprehensive investigation evaluated the potential applications of green‐synthesized ZnO nanoparticles (R‐ZnO‐NPs) and their conventional counterpart (Raw‐ZnO‐NPs) in agriculture, specifically focusing on wheat growth and antibacterial properties. The nanoparticles were synthesized using rosemary extract, which acted as a multifunctional agent for capping, stabilizing, and reducing ZnO‐NPs. These nanoparticles were also incorporated into hydrogel networks to evaluate their combined impact on plant germination, growth, and development.

SEM images revealed the triangular structure of ZnO‐NPs created through rosemary extract, showcasing uniform distribution. FTIR analysis confirmed the presence of functional groups associated with ZnO‐NPs and highlighted the complex nature of the biomolecules involved in the green synthesis. Green synthesized R‐ZnO‐NPs and R‐ZnO‐doped hydrogels did not have any significant differences in germination percentage. The shoot elongation was not affected by R‐ZnO‐NPs at almost all concentrations, while it decreased with the treatment of hydrogels. The root elongation results showed a decreasing tendency with the treatment of ZnO‐NPs suspension, similar to hydrogel treatment, compared to control. Other growth parameters also did not differ from the control groups for the treatment of foliar and hydrogels; however, the plant biomass and Zn uptake by wheat were higher than control.

When comparing the foliar application and hydrogel application methods of ZnO‐NPs synthesized in this study, it was determined that especially Raw‐ZnO‐NPs varied in chlorophyll content depending on the application type. This result indicates that wheat plants benefit more from ZnO‐NPs via the foliar application method. Additionally, antibacterial tests on E. coli and S. aureus bacteria showed that gram‐negative strains were more sensitive to R‐ZnO‐NPs than gram‐positive strains.

Our findings indicate that R‐ZnO‐NPs are more effective in promoting plant growth and exhibiting antibacterial activity against E. coli. R‐ZnO‐NPs significantly increased root and shoot elongation, likely due to enhanced uptake of ZnO nanoparticles by plant cells and activation of biochemical pathways supported by zinc's critical roles in plant metabolism. ZnO nanoparticles support plant growth by releasing zinc ions that promote cell division and expansion. R‐ZnO‐NPs exhibited higher antibacterial activity against E. coli, which can be explained by the synergistic effect of phenolic compounds present in rosemary extract and ZnO nanoparticles, disrupting bacterial cell walls and inhibiting cellular metabolism. R‐ZnO‐NPs are believed to exhibit bactericidal effects by damaging bacterial cell membranes and increasing the production of intracellular reactive oxygen species (ROS).

This research contributes valuable insights into the application of green‐synthesized ZnO‐NPs in agriculture, showcasing their multifaceted impacts on plant growth and antibacterial activities. Further studies can explore the underlying mechanisms of these effects, optimize application methods, and assess long‐term implications on soil health. The demonstrated antibacterial potential suggests applications in crop protection. Overall, this study paves the way for the development of sustainable and effective protocols for utilizing ZnO‐NPs in agricultural practices.

## 4 Experimental Section

### Materials

The ethanol (C_2_H_6_O), zinc acetate dihydrate (Zn(CH_3_COO)_2_.2H_2_O, >%98), and sodium hydroxide (NaOH) used in the ZnO NPs synthesizes were purchased from Sigma–Aldrich. All the other chemicals were in analytical grade. The rosemary (*Rosmarinus officinalis* L.) plants and wheat seeds (*Triticum aestivum*‐İkizce 96) were obtained from the market from Mersin province of Türkiye.

### Preparation of the Plants Extract and Green Synthesis of ZnO‐NPs

The rosemary (*Rosmarinus officinalis* L.) leaves were used for the synthesizes of ZnO‐NPs via green approach. To obtained plant extract, plants were first washed with tap water twice to remove any dust or other impurities and then with distilled water. The 30 g of clean rosemary leaves were placed in a conical flask and 450 mL of double‐distilled water were added and then put on a hot plate (80 °C) for 30 min. After that the prepared solution was cooled to room temperature and then the milky brown extract was filtered twice using Whatman No.1 filter papers to remove residues. The obtained extracts were stored in a refrigerator for later use.

ZnO nanoparticles can be prepared from different precursor ZnO salts. The shape, structure, optical properties and dimensions of the synthesized ZnO‐NPs may vary depending on the properties of the precursors used.^[^ ^110^ ^]^ When the literature studies were examined, it was determined that the synthesis of efficiency and high‐purity ZnO‐NPs was not sufficient when precursor salts such as zinc chloride and zinc sulfate heptahydrate were used. However, it has been observed that quite accurate and efficient results are obtained when zinc nitrate and zinc acetate precursors are used.^[^ ^110^, ^111^, ^112^ ^]^ Thus, zinc acetate dihydrate salt was used in this study as the source of Zn^2+^ cations. Green syntheses of ZnO‐NPs produced in this study were carried out according to the reference.^[^ ^113^ ^]^ Briefly, as a stock solution the 33.33 g of the zinc acetate dihydrate salt was dissolved in 1000 mL distilled water. Then 90 mL of the plants’ extract was added to 150 mL of stock solution and the mixture was vigorously stirred for 10 min. After this time, the 1 M NaOH was dripped in the mixture till the pH value reach 12.0. The solution was continuously stirred (100 rpm) at room temperature for 2 h to produce ZnO‐NPs. Then the mixtures were rested until the sedimentation process finished and precipitate obtained by filtration washed with deionized water repeatedly. Finally, the precipitate was dried in an oven overnight at 60 °C. The milky brown powder of green synthesized ZnO‐NPs was ground into a fine powder, weighed and stored for the characterization. For the Raw ZnO‐NPs production, the same process was applied except the addition of plant extracts.

### Synthesize of ZnO‐NPs Doped Hydrogel

To use in the plant experiments, the synthesized ZnO‐NPs powders (with standard techniques‐ Raw‐ZnO‐NPs and using with rosemary extract via green route‐ R‐ZnO‐NPs) added to SA and PVA hydrogels solutions. Briefly, 3 g polyvinyl alcohol (PVA) and 2 g sodium alginate (SA) were dissolved in 100 mL distillated water and the mixture was heated 95 °C for 1 h. Then this mixture was cooled to room temperature and synthesized ZnO‐NPs powder at different concentrations (100–500 mg to 100 mL) were added to the mixture while it was stirred at 120 rpm. The synthesized ZnO‐NPs doped hydrogels will hereinafter be expressed as 0.1%−0.5% (for example, 100 mg R‐ZnO‐NPs added to 100 mL PVA/SA solution as 0.1% R‐ZnO‐HG). This solution was dripped with a syringe into CaCl_2_ (5% w/v) solution stirred at 100 rpm and the produced hydrogels beads were kept in the CaCl_2_ solution for a night. The same procedure was applied for the raw hydrogel beads except ZnO‐NPs addition. Finally, the obtained Raw and ZnO‐NPs‐doped hydrogel beads were washed with deionized water to remove residual CaCl_2_. The beads were stored in a refrigerator for plants experiments.

### Swelling Properties of Synthesized Hydrogels in Aqueous and Soil Media

The swelling properties in aqueous media of synthesized raw hydrogels (Raw‐HG), ZnO‐NPs‐doped hydrogel (Raw‐ZnO‐HG) and synthesized ZnO‐NPs using rosemary extract doped hydrogel (R‐ZnO‐HG) were determined according to reference.^[^ ^114^ ^]^ For that, synthesized hydrogel beads were left to dry for 4 days at room temperature. Then, 0.2 g of dried hydrogels (w_dry_) were left in tap water (pH 7.68) at room temperature for 4 days until the equilibrium swelling capacity. The swelled hydrogel beads were removed from the water, slightly dried with wet filter paper, and weighed immediately (w_swelled_). All treatments were conducted in 3 replicates. The equilibrium swelling capacity was calculated using Equation 1:

(1) $$ESC\;\left(\%\right)=\frac{\left(Wswelled-Wdry\right)}{Wdry}\;x100$$

In addition to the swelling capacity in free water system, the swelling experiments were also conducted in soil‐water mixture media. The soil was used in the experiments and the properties of the soil were pH:7.37, electrical conductivity (EC): 628 µs cm^−1^, organic matter content (OMC) 1.21%, lime 24%, clay (< 0.002 mm) 1.3%, silt (0.002–0.06 mm) 10%, sand (0.06–2 mm) 88%, saturation degree (SD) 33%, and salt 0.012 dS m^−1^. Briefly, the 100 g loamy sand soil samples, 1 g hydrogels, and 1 g hydrogel+100 g soil were weighed and put into permeable nylon bags. Then the bags were immersed in glass beaker filled with two liter of tap water (pH 7.68) for 15 min.^[^ ^67^ ^]^ The swelled hydrogel beads, wet soil, and the mixture were removed from the water, slightly dried with wet filter paper, and weighed immediately. All treatments were conducted in 3 replicates. The swelling capacity of hydrogels in soil‐water media was calculated as follow Equation 2.

(2) $$SC=\frac{\left(Wwet\;hydrogel+soil\;-\;Wwet\;soil\right)}{Wdry\;hydrogel\;in\;mixture}$$

### Characterization Techniques

The morphology of ZnO‐NPs was studied using scanning electron microscopy (SEM, FEI, Quanta FEG 250) with Cu‐K radiation. To ascertain the chemical composition of the ZnO‐NPs and the purity of the phase, X‐ray Diffraction Spectroscopy (XRD, Bruker, D8 Advance) with a 2 scan was utilized. Using the Debye‐Scherer equation (Equation 3), the average size of produced ZnO NPs was also determined. A Bruker Alpha spectrometer was used to perform Fourier transform infrared (FT‐IR) spectroscopy spanning the wavelength range of 400–4000 cm^−1^ to examine functional groups of ZnO‐NPs. The powder samples were analyzed with the KBr pellet method.

(3) $$D\;=\frac{0.9\lambda }{\beta \;Cos\theta }$$

The D corresponding to the size of the crystalline grains was calculated using the Debye‐Scherrer equation. Where, β (in rad) is the full width at half‐maximum (FWHM) of the XRD peak, λ is the wavelength of x‐rays, θ is the angle at which the observed peak, and k is the constant value (k = 0.9).^[^ ^115^ ^]^

### Preparation of ZnO‐NPs Suspensions and Seed Germination Test

Green synthesized ZnO‐NPs suspensions at different concentrations (1000, 2000, 3000, 4000, and 5000 mg L^−1^) were prepared by using deionized water. The ultrasonication process were employed to complete homogenization of ZnO‐NPs in solution, through 30 min at 25 °C. The pH values of Raw‐ and R‐ZnO‐NPs suspensions was determined ≈8.50 and 8.80, respectively. The uniform wheat seeds (*Triticum aestivum*‐ İkizce 96) were selected to minimize error in germination tests and they were sterilized with 70% ethanol for 30 s. After the ethanol sterilization, the seeds were washed with distillated water 5 times for 5 min. The double layer filter paper used as an inert material cut and put into the petri dishes (100×20 mm), and 10 wheat seeds were placed on the paper. After the ultrasonication, 5 mL suspension at different concentration of ZnO‐NPs synthesized with rosemary (R‐ZnO‐NPs) extract or Raw‐ZnO‐NPs were added to petri dishes. The petri dishes were put into an incubator for 7 days at 25 °C in dark. For the control groups, the seeds were exposed to 5 mL distillated water. In addition to, 3 mL distillated water and 2 g of the synthesized R‐ZnO‐NPs‐HG and raw hydrogels (swelled) were added to petri dishes to determine the effective of hydrogels on wheat seed germination process. All the treatments were conducted in three replicates and completely randomized designed. After the seed germination process, the number of germinated seeds were counted, and the radicle and plumule length was measured using millimetric paper.^[^ ^116^ ^]^ The germination percentage and the seedling vigor index was calculated using Equation 4.

(4) $$Seedling\;vigor\;index\;\;\left(SVI\right)=GP\left(\%\right)x\;\left(RL+PL\right)$$

where GP means germination percentage (%), RL means radicle length (cm), and PL means plumule length (cm).

### Pot Experiments

Both pot experiments (treatments of suspension and hydrogel) were conducted in pots contained 80 g peat in the laboratory conditions at room temperature (25 °C) at sowing time. The completed surface sterilized (sterilization process was mentioned in above Section) 5 wheat seeds were sown in pots and watered with tap water (pH 7.2 and conductivity 107.9 mScm^−1^). The foliar treatment procedure was conducted according to.^[^ ^117^ ^]^ ZnO‐NP suspensions prepared at different concentrations (1000, 2000, 3000, 4000, and 5000 mg L^−1^) were applied to the plants by foliar spraying after ultrasonication. The ZnO‐NPs concentrations selected here were determined based on the literature studies.^[^ ^91^, ^118^ ^]^ After the germination period (7 days), the first foliar treatment was applied with a hand sprayer. Before the foliar application, the turf surface was covered with foil to prevent contamination of the turf. The foliar spraying applications were done every seven days for a total of three applications. Total quantity of synthesized Raw‐ and R‐ZnO‐NPs used per pot were 15 mL. Besides that, the other treatment was hydrogel application. In accordance with, 2 g the synthesized Raw‐HGs, Raw‐ZnO‐HGs, and R‐ZnO‐HGs were added to pots which included 5 wheat seeds and watered with tap water (pH 7.68 and conductivity 108.9 mS cm^−1^). The ZnO‐NPs content in hydrogels were 0.2% (w/v) for all the types of hydrogels based on the results of swelling capacity, except Raw‐HG. The wheat seedlings were grown for 21 days after germination period, and the chlorophyll content was measured with SPAD‐502 chlorophyll meter (Konica–Minolta, Japan, 0.06 cm^2^ measurement area). It was given as SPAD value. The plant heights, fresh weights, dry weights of seedling plants and Zn content were determined. To determine the Zn content in plants, plants were harvested, shoot parts were weighed (as fresh weight‐FW) and left in an oven to reach constant weight (DW) for 2 h at 65 °C. The dried plants were digested in 5 mL 12 M HNO_3_ on a hot plate at 220 °C.^[^ ^119^ ^]^ The Zn concentration of plants was determined by inductively coupled plasma (ICP‐MS) (Agilent 7500ce Model).

### Antimicrobial Activity

The agar well diffusion method was used to determine the antibacterial effects of synthesized Raw‐ZnO‐NPs and R‐ZnO‐NPs on *Staphylococcus aureus* (gram‐positive) and *Escherichia coli* (gram‐negative) bacteria. The bacterial colonies were first inoculated to a Tryptic Soy Broth (TSB) and cultured for 24 h at 37 °C. After the incubation process, *S. aureus* and *E. coli* culture was transferred to fresh TSB tubes and incubated at 37 °C for 5 h and then the absorbance of the bacteria was determined at 625 nm (≈10^6–7^ cfu mL^−1^). The spread plate technique using Triptic Soy Agar (TSA) was applied. When the TSA was solidified, 0.1 mL of bacterial cultures were spread homogenously. Wells with a diameter of 10 mm were created using sterile tubes, and 100 µL of suspensions were placed inside each well. The solutions were included synthesized Raw‐ZnO‐NPs or R‐ZnO‐NPs at different concentrations (0.01, 0.02, 0.04 g mL^−1^).^[^ ^5^ ^]^ DMSO (dimethyl sulfoxide) is an effective solvent to hinder the aggregation of ZnO‐NPs. The negative control (NC) and positive control (PC) were realized using with DMSO (10%) and antibiotic (Amoxicillin, 50 µg), respectively. The petri dishes were left for the incubation at 37 °C for a night and the diameters around the wells were measured via Vernier caliper.^[^ ^10^ ^]^ All the antibacterial tests were performed in triplicate.

### Statistical Analyses

All the treatments were realized in three replicates. Statistical analysis was performed by one‐way analysis of variance (ANOVA) using the statistical software SPSS version 20 (IBM Inc., New York, USA). The means were compared using Post Hoc least significant difference (LSD) test and the significance level is 5%.

## Conflict of Interest

The authors declare no conflict of interest.

## Data Availability

The data that support the findings of this study are available from the corresponding author upon reasonable request.
